# The Role of Vitamins in Pediatric Urinary Tract Infection: Mechanisms and Integrative Strategies

**DOI:** 10.3390/biom15040566

**Published:** 2025-04-11

**Authors:** Joanna Wróblewska, Hanna Złocińska, Marcin Wróblewski, Jarosław Nuszkiewicz, Alina Woźniak

**Affiliations:** 1 Department of Medical Biology and Biochemistry, Faculty of Medicine, Ludwik Rydygier Collegium Medicum in Bydgoszcz, Nicolaus Copernicus University in Toruń, 24 Karłowicza St., 85-092 Bydgoszcz, Poland; joanna.wroblewska@cm.umk.pl (J.W.); jnuszkiewicz@cm.umk.pl (J.N.); 2 Brudziński Provincial Children’s Hospital, 44 Chodkiewicza St., 85-667 Bydgoszcz, Poland; zlocinskahanna@gmail.com

**Keywords:** antioxidants, antimicrobial defense, oxidative stress, pediatric urinary tract infections, uropathogens, vitamin supplementation, vitamins

## Abstract

Urinary tract infections (UTI) are among the most frequent bacterial infections in children, representing a significant cause of morbidity with potential long-term complications, including renal scarring and chronic kidney disease. This review explores the multifaceted roles of vitamins A, D, E, and C in the prevention and management of pediatric UTI. Vitamin A supports mucosal barrier integrity and immune modulation, reducing pathogen adhesion and colonization. Vitamin C exhibits antioxidant and antimicrobial properties, acidifying urine to inhibit bacterial growth and enhancing the efficacy of antibiotics. Vitamin D strengthens innate immunity by promoting antimicrobial peptide production, such as cathelicidins, and improves epithelial barrier function, while vitamin E mitigates oxidative stress, reducing renal inflammation and tissue damage. The interplay between oxidative stress, immune response, and nutritional factors is emphasized, highlighting the potential of these vitamins to restore antioxidant balance and prevent renal injury. Complementary strategies, including probiotics and phytotherapeutic agents, further enhance therapeutic outcomes by addressing microbiome diversity and providing additional antimicrobial effects. While these approaches show promise in mitigating UTI recurrence and reducing dependence on antibiotics, evidence gaps remain regarding optimal dosing, long-term outcomes, and their integration into pediatric care. By adopting a holistic approach incorporating vitamin supplementation and conventional therapies, clinicians can achieve improved clinical outcomes, support antibiotic stewardship, and reduce the risk of renal complications in children with UTI.

## 1. Introduction

Urinary tract infections (UTIs) are a common health concern, affecting different demographics with varying prevalence. The incidence of UTI in children under 3 years of age in the United States is estimated at 400,000 cases per year [1]. UTIs occur in any part of the urinary tract and can spread from the urethra to the bladder and even the kidneys [2]. UTI can be classified according to the extent of urinary tract involvement, typically into cystitis (infection confined to the bladder) and pyelonephritis (involvement of the kidneys). This distinction is critical, as pyelonephritis carries a higher risk of long-term complications, including renal scarring and chronic kidney disease [3]. During the first year of life, UTIs are more common in boys. However, after the first year, the prevalence shifts, and girls are much more likely than boys to develop UTI [1,4,5]. Febrile UTIs are of particular concern, as they are strongly associated with renal scarring, especially when diagnosis or treatment is delayed, and the risk increases with recurrent episodes [6]. Several risk factors significantly contribute to the occurrence of UTI in children. Congenital anomalies of the urinary tract, such as vesicoureteral reflux and posterior urethral valves, remain major contributors [2,6]. Additionally, bladder bowel dysfunction (BBD), which disrupts normal urinary flow, is another important factor [7]. Obesity has also been linked to an increased risk of lower urinary tract dysfunction in children. This includes symptoms such as incontinence and urgency, which may predispose affected children to a higher prevalence of urinary tract infections by altering normal bladder function and voiding patterns [8,9]. Constipation is another notable risk factor that exacerbates BBD and contributes to recurrent infections [6,10].

UTI develops when bacteria enter the urinary tract lumen and multiply, causing inflammation. Routes of infection include the urethra, gastrointestinal tract, bloodstream, and lymphatic system [11]. The most common pathogen responsible for UTI in children is *Escherichia coli*, which accounts for 80–90% of cases, making it the predominant causative agent of these infections [11,12]. Other common uropathogens include *Klebsiella pneumoniae*, *Proteus mirabilis*, *Pseudomonas aeruginosa*, *Enterococcus faecalis*, and, less frequently, *Staphylococcus aureus* and *Citrobacter* spp. [2,10,11,12,13,14]. Advanced research on the urinary tract microbiome highlights the critical balance between commensal and opportunistic pathogens in maintaining urinary tract health [15]. Emerging evidence suggests that microbiome diversity plays a crucial role in preventing UTI recurrence, as a balanced microbiome supports urinary tract health [16,17]. Reduced microbiome diversity, including the loss of *Lactobacillus* spp. and *Bifidobacterium* spp., correlates with increased *E. coli* colonization and higher rates of UTI recurrence [18,19,20]. Catheter-associated urinary tract infections (CAUTI) are a significant complication of hospital care, particularly in pediatric patients. CAUTI are typically polymicrobial, with Gram-positive uropathogens, such as *E. faecalis* and coagulase-negative staphylococci, playing a significant role alongside Gram-negative bacteria, such as *E. coli* and *P. mirabilis* [21]. The formation of biofilms on catheter surfaces not only enhances bacterial survival and resistance to antibiotics but also limits antibiotic penetration, facilitates the exchange of resistance genes between microorganisms, and enables bacteria to evade host immune defenses, significantly complicating the effective treatment of infections [21,22]. Pathogens such as *S. aureus*, *Staphylococcus epidermidis*, *Staphylococcus haemolyticus*, *Staphylococcus lentus*, and *E. faecalis* are known for their strong biofilm-forming capabilities, which significantly hinder their elimination and complicate infection management. Additionally, other species, including *E. coli*, *Klebsiella* spp., *Proteus* spp., *Providencia* spp., and *Pseudomonas aeruginosa*, have also been identified as strong biofilm producers, further contributing to the difficulty of managing these infections [22,23].

Recent findings highlight the critical involvement of oxidative stress (OS) in the pathogenesis of UTI, particularly in pediatric cases. OS arises from an imbalance between reactive oxygen species (ROS) generation and the cells’ antioxidant defenses. Elevated ROS levels, including hydroxyl radicals (^•^HO) and superoxide anions (O_2_^−^), are a common response during infections and contribute to lipid peroxidation, as indicated by increased levels of malondialdehyde (MDA). Conversely, total antioxidant capacity, a measure of the body’s ability to counteract oxidative damage, is significantly reduced during UTI. This imbalance exacerbates tissue damage in the urinary tract, particularly the kidneys, and may increase the severity of infections and the risk of renal scarring [24,25,26,27]. The inflammatory response further amplifies renal tissue damage. Activated inflammatory cells, such as neutrophils and macrophages, release ROS, which contribute to oxidative renal injury, fibrosis, and scarring. For example, neutrophils generate superoxide anions and hydrogen peroxide during the respiratory burst, while macrophages produce nitric oxide and peroxynitrite, exacerbating tissue damage and promoting chronic inflammation. These processes are linked to the release of pro-inflammatory and proteolytic enzymes that exacerbate tissue damage [24,25,28,29]. Monitoring OS markers, such as total antioxidant status (TAS) and oxidative stress index (OSI), provides valuable insights into the severity of inflammation and the recovery trajectory. Prolonged inflammation, reflected by higher TAS and lower OSI values, indicates ongoing OS and the need for therapeutic interventions. The vitamins A, C, D, and E are essential antioxidants that play a significant role in maintaining the oxidant–antioxidant balance, thereby mitigating oxidative stress and its harmful effects [30,31,32]. Interventions aimed at restoring the oxidant–antioxidant balance, such as antioxidant therapies, could complement antibiotic regimens and help protect pediatric kidney health [26,33].

Despite advancements in understanding the pathophysiology and management of UTI in pediatric populations, significant gaps remain in leveraging nutritional interventions, particularly vitamins, to mitigate infection risks and long-term renal complications. This review aims to bridge these gaps by providing a comprehensive synthesis of current research on the roles of vitamins A, D, E, and C, which are currently the only vitamins with demonstrated roles in preventing and managing pediatric UTI. This review aims to bridge these gaps by providing a comprehensive synthesis of current research on the roles of vitamins A, D, E, and C in preventing and managing pediatric UTI. By exploring the mechanisms underlying their protective effects, such as enhancing immune responses, maintaining epithelial integrity, and mitigating OS, this article seeks to highlight their therapeutic potential. Additionally, the review examines emerging evidence on complementary strategies, such as probiotics and phytotherapeutic agents, which could synergize with vitamin-based interventions.

## 2. The Role of Vitamin A in Enhancing Immunity and Preventing Pediatric Urinary Tract Infections

Vitamin A has been recognized as an anti-infective vitamin since the 1920s [34]. It comprises a group of essential unsaturated organic compounds, including retinol, retinal, retinoic acid, and provitamin A carotenoids like beta-carotene [35,36]. Vitamin A plays a crucial role in immunity by supporting mucosal barriers, antibody responses, and the functionality of lymphocytes, including T- and B-cells. Its deficiency mirrors immunodeficiency, impairing immune function and increasing susceptibility to infections, particularly those targeting mucosal surfaces like the urinary tract [34,36,37]. Retinol, acting as a hormone-like growth factor, promotes the re-epithelialization of damaged mucosal surfaces, restoring the integrity of the urinary tract and reducing pathogen adhesion and colonization [37]. Reducing uropathogens adhesion and colonization is essential in preventing renal scarring and mitigating the severity of infections [34,35,38]. Studies show that vitamin A supplementation supports repair mechanisms, strengthens mucosal defenses, and supports the effectiveness of the immune system in maintaining urinary tract sterility by enhancing the immune response and epithelial integrity, which prevents bacterial growth [39,40]. Additionally, experimental studies on animal models confirm the protective role of vitamin A in oxidative damage and kidney function. Salehzadeh et al. [41] demonstrated that vitamin A supplementation reduces OS markers like MDA and improves renal histology under toxic conditions. Similarly, Kanter et al. [42] highlighted the antioxidative and nephroprotective effects of vitamin A in combination with vitamin C, showing significant decreases in renal OS and tissue damage in endotoxemia-induced kidney injury in rats. Vitamin A has also demonstrated effectiveness in reducing renal scarring in children with pyelonephritis [43]. A meta-analysis by Zhang et al. [44] provided strong evidence of a significant reduction in renal damage when vitamin A is used as an adjunct to antibiotics, further supporting its therapeutic benefits in managing acute pyelonephritis. Additionally, its anti-inflammatory properties help preserve kidney function and prevent long-term complications, such as fibrosis [45]. In a cohort study conducted in Tehran, 25 children with proven UTI and 40 healthy controls were compared for serum vitamin levels. While no statistically significant difference was found in vitamin A levels between the groups, nutritional deficiencies, including vitamin A deficiency, were associated with an increased risk of recurrent UTI in children [38]. Animal model studies further support the role of vitamin A in mitigating renal OS and maintaining structural integrity of kidney tissues under conditions of oxidative damage or toxicity, suggesting its broader therapeutic potential [41,42]. Regional dietary habits and socioeconomic conditions appear to influence vitamin A status in pediatric populations, making targeted interventions crucial in resource-limited settings. Vitamin A supplementation demonstrates substantial promise in preventing and managing UTI [40,41,42,43,45]. While the evidence supports its utility, further studies are necessary to refine dosing strategies and evaluate its broader applicability.

## 3. The Role of Vitamin C in Antioxidant Defense and Adjunctive Therapy for Pediatric Urinary Tract Infections

Vitamin C (ascorbic acid), discovered in the early 20th century as a cure for scurvy, has since been associated with protective effects against infections, including pneumonia and UTI, due to its antioxidant properties. Vitamin C supports the immune system through collagen synthesis and antioxidant activity [14,46]. Moreover, vitamin C contributes to immune modulation by enhancing innate immune responses, including neutrophil chemotaxis, phagocytosis, and ROS production, which are critical for effective pathogen elimination [14,47]. Its role in preventing recurrent UTI is linked to two mechanisms: acidifying urine to inhibit bacterial growth and increasing the production of reactive nitrogen species, including nitric oxide (NO). Acidified, nitrite-rich urine promotes NO production, which is toxic to pathogens such as *E. coli*, *P. aeruginosa*, and *Staphylococcus saprophyticus*. Vitamin C enhances this effect by shifting biochemical reactions toward greater NO production, providing an effective mechanism for bacterial growth inhibition [13,46,48,49]. Prior UTI or conditions promoting urinary stasis increase the risk of stone formation in the pediatric population [50]. For struvite stones, this phenomenon is almost exclusively associated with the presence of urease-producing bacteria, including *P. aeruginosa*, *P. mirabilis*, and *Klebsiella pneumoniae* [51]. Research conducted by Manzoor et al. [52] demonstrated that vitamin C reduces the size, growth rate, and formation of struvite crystals induced by uropathogenic *P. aeruginosa*. Vitamin C also influences calcium oxalate crystal formation in urine by lowering urinary pH. However, this effect is insufficient in conditions like idiopathic hypercalciuria, where calcium oxalate crystals damage renal epithelium and increase infection risk [53].

Management of UTI in children involves both nitrofurantoin, fosfomycin, and trimethoprim-sulfamethoxazole are commonly recommended for uncomplicated UTI, while aminoglycosides and carbapenems are reserved for complicated or multidrug-resistant cases [54,55,56]. It has been demonstrated that vitamin C shows a synergistic effect when combined with ciprofloxacin, nitrofurantoin, sulfamethoxazole, and gentamicin against *E. coli* strains resistant to these drugs [57,58,59]. Hassuna et al. [60] demonstrated that vitamin C not only enhances the efficacy of antibiotics but also compromises the integrity of bacterial membranes and disrupts bacterial cell walls, making bacteria more susceptible to antimicrobial agents. These effects were observed both in vitro and in vivo, where the combination of vitamin C with antibiotics significantly enhanced treatment effectiveness. Furthermore, vitamin C induces oxidative stress within bacterial cells, which potentiates the bactericidal effects of antibiotics without diminishing their efficacy. These findings underscore the potential of vitamin C as an effective adjunctive therapy in managing resistant uropathogenic *E. coli* infections.

Vitamin C influences the initial stage of bacterial biofilm formation by reducing the adhesion capacity of uropathogens to surfaces, making it a promising adjunct in managing catheter-associated infections [61]. Studies by Amábile-Cuevas [58] demonstrated that vitamin C, at concentrations attainable in urine, effectively inhibits the growth of antibiotic-resistant *E. coli* strains cultured in synthetic human urine. This effect was attributed to its ability to acidify the environment and enhance OS within bacterial cells. Urease-positive bacteria like *P. mirabilis* can counteract its acidifying effects by alkalizing the urine, which may limit its intended benefits in managing catheter-associated infections [62,63]. Low concentrations of vitamin C (below 3.12 mg/mL) have been found to increase biofilm production by providing a potential nutrient source for bacteria, highlighting the importance of using appropriately high doses (equal to or greater than 6.25 mg/mL) to achieve its anti-biofilm effects. In the presence of fluoroquinolones, vitamin C at a concentration of 0.4 mg/mL in urine may promote biofilm formation by *P. mirabilis* strains [64]. An alternative solution could be using vitamin C derivatives in the form of lipophilic esters, such as ascorbyl-6-*O*-alkanoates, which exhibit excellent stability and potentially more potent antibacterial activity, including against resistant bacterial strains that readily form biofilms. The nanostructures they form facilitate diffusion through the biofilm matrix while also enabling the elimination of persistent bacterial cells by altering membrane flexibility and inducing local redox activity [65]. Phenolic esters of ascorbic acid are lipophilic derivatives of vitamin C, exhibiting enhanced antioxidant and protective properties, particularly in shielding lipid membranes from oxidative stress. These properties may contribute to increased resistance of epithelial cells to oxidative damage, which could be beneficial in protecting the urinary tract epithelium during UTI. However, direct evidence supporting antimicrobial activity in this context remains to be further explored [66]. The increasing antibiotic resistance may require additional therapeutic strategies, such as the use of vitamin C derivatives, which support the protection of the urinary tract epithelium and reduce the effects of oxidative stress caused by infection [61].

In conclusion, while vitamin C shows promise as a supportive therapy for UTI and other infections due to its antioxidant and antimicrobial properties, its limitations, including potential interactions with specific antibiotics and variability in effectiveness, must be carefully considered.

## 4. Vitamin D and Pediatric Urinary Tract Infections

Vitamin D deficiency is a significant risk factor for urinary tract infections, affecting 45.93% of children diagnosed with this condition [67,68,69]. The active form of vitamin D, 1,25-dihydroxyvitamin D3 (calcitriol), synthesized primarily in the kidneys and locally in macrophages during infection, modulates the immune response by enhancing the production of antimicrobial peptides such as cathelicidins (LL-37) and β-defensins and regulating cytokine activity [2,69,70]. Reduced levels of cathelicidin during UTI result in increased bacterial persistence and a higher likelihood of recurrent infections, especially in children with chronic vitamin D deficiency [71,72]. The development of UTI in children is closely associated with significantly reduced levels of 25-hydroxyvitamin D (25-OH-vit D) in serum, which is a key marker of vitamin D status. This reduction is often accompanied by an increase in the level of vitamin D binding protein, which may further decrease the bioavailability of active vitamin D. Increased vitamin binding protein levels may enhance immune dysregulation, affecting the bioavailability of active vitamin D and impairing the antimicrobial response [73].

Low serum vitamin D levels are associated with an increased risk of recurrent UTI in children, as confirmed by the study by Merrikhi et al. [74]. This study included 68 children around six years of age. The study group was divided into an intervention group receiving 1000 IU of vitamin D daily for six months and a placebo group. While serum cholecalciferol (vitamin D3) levels increased significantly in the intervention group, there was no significant reduction in the rate of recurrent urinary tract infections. The authors suggested that higher doses or longer supplementation may be necessary to observe protective effects. Moreover, research by Seifollahi et al. [75] highlighted that low serum levels of both vitamin D and zinc are common in children with UTI. This suggests that deficiencies in these nutrients may synergistically compromise immune defenses, increasing susceptibility to infections. The combined supplementation of vitamin D and zinc could, therefore, represent a promising approach to enhancing immune mechanisms and potentially reducing the risk of recurrent UTI in children. Population studies reveal variability in the effects of vitamin D. Clinical data indicate that vitamin D deficiency is an independent risk factor for renal scarring in children with recurrent urinary tract infections. In patients with such infections, low levels of 25(OH)D were significantly more frequently observed in the group with scarring lesions detected on renal scintigraphy [76]. Other studies confirm that children with renal parenchymal damage or recurrent urinary tract infections had lower vitamin D levels than control groups [77,78]. A meta-analysis of 839 children with UTI showed that children with serum vitamin D levels below 20 ng/mL were more likely to develop UTI. This association was stronger in Asian populations and in female children [79]. Children with serum 25(OH)D levels below 20 ng/mL have been shown to be at a higher risk of developing UTI compared to children with normal vitamin D levels [80]. Contrary to previous findings, some studies suggest that vitamin D supplementation may increase the risk of UTI in formula-fed infants under 3 months of age, with a reported risk increase of up to 76%. This effect may be attributed to the potential development of mild nephrocalcinosis, which creates a favorable environment for bacterial growth. Furthermore, vitamin D, as an immune modulator, may paradoxically suppress immune responses or disrupt the local conversion of 25(OH)D to its active form, 1,25-dihydroxyvitamin D, at infection sites, potentially heightening susceptibility to UTI. Excessive concentrations of 25(OH)D may also act antagonistically to 1,25-dihydroxyvitamin D, further dysregulating immune responses. Therefore, while vitamin D deficiencies are commonly associated with an increased risk of infections, the use of supplementation should be approached cautiously, taking into consideration the patient’s age, appropriate dosing, and individual risk factors to prevent undesirable effect [81,82].

There may be an association between vitamin D status and the distribution of specific UTI pathogens in children. Regardless of vitamin D levels, the major etiologic factor remains *E. coli*, accounting for 47.61% of cases in children with mild vitamin D deficiency and 56.36% in children with moderate deficiency. Variations in vitamin D levels have been linked to UTI susceptibility, and multiple pathogens, including *Klebsiella* spp., *Pseudomonas* spp., *Enterococcus* spp., and *S. aureus*, have been identified in affected children, suggesting a broader role for vitamin D in immune function [67]. The results of study by Mohanty et al. [83] showed that during *E. coli* infection, vitamin D significantly increases the expression of occludin and claudin-14 in the superficial layers of the bladder epithelium. Vitamin D may contribute to the strengthening of the bladder lining by supporting epithelial integrity and potentially reducing bacterial adhesion, as suggested by its role in enhancing immune defenses and modulating host responses during infection [68,84]. Recent studies have also shown that vitamin D has significant antibiofilm activity against biofilm-forming pathogens such as *K. pneumoniae* and *P. aeruginosa*. This property increases its potential use in treating recurrent or antibiotic-resistant UTI by targeting biofilm structures that often complicate the treatment of infections [85]. Despite the immense potential of vitamin D in antimicrobial defense, there are also limitations. For example, a lack of significant synergy between vitamin D and antibiotics such as piperacillin/tazobactam and doripenem has been observed against multidrug-resistant bacteria like *Acinetobacter baumannii* [40]. Due to the observed variability in the effects of vitamin D supplementation, especially in infants and children, it is crucial to precisely adjust the dosage according to age and body weight and monitor safety during use. According to current recommendations, the optimal and safe prophylactic dose of vitamin D is 400 IU/day for infants aged 0–6 months, 400–600 IU for children aged 6–12 months, 600–1000 IU for children aged 2 to 10 years, and 800–2000 IU per day for adolescents aged 11–18 years, depending on diet and sun exposure [86]. Increasing vitamin D supplementation doses leads to a significant rise in 25(OH)D levels but does not always result in additional health benefits and may increase the risk of exceeding the safe concentration range. Although vitamin D toxicity is rare, cases of hypercalcemia and nephrocalcinosis have been reported, particularly with long-term use of high doses or the intake of unregulated supplements obtained outside pharmaceutical supervision [87].

Despite the established correlation between vitamin D deficiency and increased susceptibility to UTI, there is limited research exploring the specific effects of vitamin D supplementation in pediatric populations. Most available data focus on adult populations, leaving significant gaps in understanding its efficacy and dosage for children.

## 5. The Protective Role of Vitamin E in Pediatric Urinary Tract Infections

Vitamin E includes a group of structurally similar compounds categorized into tocopherols and tocotrienols, with tocotrienols exhibiting distinct properties beyond those of tocopherols, such as enhanced neuroprotection, cholesterol-lowering, and potent antioxidant effects. Tocotrienols are more effective than tocopherols in specific cellular contexts due to their unique ability to penetrate saturated fatty layers of cell membranes, including the brain and liver [88]. Vitamin E has been demonstrated to act synergistically with other antioxidants, such as epicatechin, enhancing its protective effects against OS [89]. Vitamin E, as a fat-soluble antioxidant, plays a protective role by reducing oxidative damage, which is particularly relevant in minimizing renal scarring in children with acute pyelonephritis. This effect is primarily attributed to its ability to neutralize ROS and support cellular repair mechanisms in the kidneys [90]. Research by Kavutcu et al. [91] highlights the critical role of vitamin E in protecting kidney tissues from oxidative damage induced by gentamicin toxicity. The study demonstrated that gentamicin significantly reduced the activity of key enzymatic antioxidants, such as superoxide dismutase and catalase, which are essential for neutralizing ROS and preventing cellular damage. Vitamin E supplementation was shown to effectively restore the activity of these enzymes, counteracting the OS caused by gentamicin. By mitigating lipid peroxidation and reactivating the antioxidant defense system, vitamin E provided substantial protection to renal tissues, emphasizing its potential as a therapeutic agent in conditions characterized by heightened OS. Ghasemi et al. [90] explored the potential role of vitamin E as an adjunct therapy in acute pyelonephritis (APN) in children. The study included 85 participants aged 3 months to 14 years who received a 4-month course of vitamin E supplementation at 20 IU/kg/day in addition to standard antibiotic treatment. While the study did not observe a statistically significant reduction in renal scarring on dimercaptosuccinic acid scans, it identified a trend toward reduced photopenic areas in specific subgroups, particularly girls and children aged 1–3 years. These findings suggest that vitamin E may have a role in reducing OS and preventing the progression of renal damage in certain pediatric groups, though larger, more comprehensive trials are needed to validate these outcomes and refine treatment protocols. Yousefichaijan et al. [92] investigated the effects of vitamin E supplementation as an adjunct treatment in a double-blinded randomized controlled trial involving 152 girls aged 5 to 12 years with their first episode of APN. Participants were divided into two groups: one receiving antibiotics alone (control group) and the other receiving antibiotics combined with 100 IU/day of vitamin E for 14 days. The study demonstrated that vitamin E significantly improved clinical symptoms of APN, such as fever, urinary urgency, frequency, and incontinence, during the acute phase of the infection. These findings suggest that vitamin E’s antioxidant properties may alleviate symptoms during the acute phase of APN but have limited long-term effects on renal inflammation or scarring. The study highlights the potential role of vitamin E in enhancing symptom management, though further research is recommended to explore its long-term benefits. The clinical efficacy of vitamin E in reducing the risk of renal scarring was also confirmed in a study by Sobouti et al. [45]. This randomized trial involving children with acute pyelonephritis showed that vitamin E supplementation combined with antibiotic therapy effectively prevented the progression of kidney lesions observed in DMSA (dimercaptosuccinic acid) scintigraphy after six months. None of the patients in the supplemented group showed worsening lesions, whereas in the control group, progression occurred in 42.5% of the children [45].

Vitamin E has shown potential to reduce biofilm formation in several pathogens. In the form of α-tocopheryl acetate, it significantly inhibits the biofilm development of *S. aureus*, *S. epidermidis*, *Pseudomonas putida*, and *P. mirabilis*. Pretreatment of silicone catheter surfaces with vitamin E reduced bacterial colonization, primarily by *S. aureus* and *S. epidermidis*. These results suggest that vitamin E coatings on medical devices may help prevent biofilm-associated infections [93]. Beyond its effects on biofilm formation, vitamin E has also been studied for its potential role in enhancing the efficacy of antibiotics used to treat bacterial infections, including UTI. Vitamin E has been shown to enhance the activity of imipenem, a drug used to treat bacterial UTI, against *A. baumannii* strains [40]. There is mixed evidence regarding the supportive role of vitamin E in the treatment of *S. aureus* infections, though not specifically in urinary tract infections. The study by Shahzad et al. [40] found no significant effect of vitamin E (tocopherol) on *S. aureus*, even when combined with linezolid. In contrast, the study by Pierpaoli et al. [94] demonstrated that tocotrienols enhanced the efficacy of daptomycin. These differences may be attributed to the use of different forms of vitamin E, similar to observations with vitamin A. Further research highlights tocotrienols as a lesser-studied but potent form of vitamin E with unique properties that extend their potential clinical applications. Unlike tocopherols, tocotrienols exhibit superior antioxidant activity and more efficient distribution in lipid-rich membranes of vital organs such as the brain and liver. These characteristics underscore their distinct advantages over tocopherols in targeted therapeutic applications, particularly in managing OS and inflammation [88].

**Figure 1:**
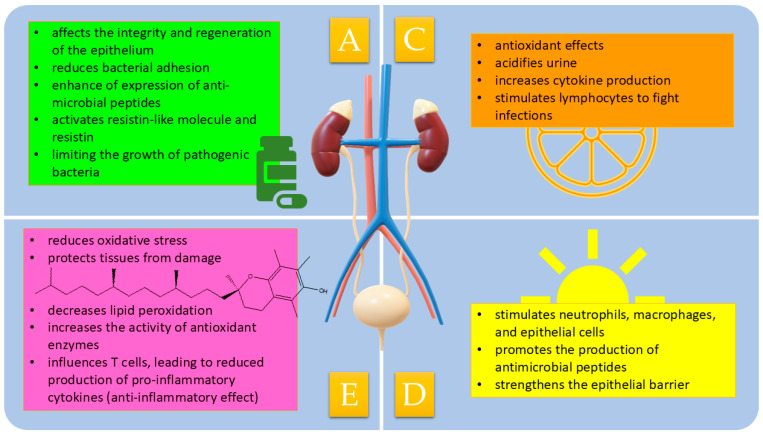
The role of vitamins A, C, D, and E in urinary tract infections.

In Figure 1, the significance of vitamins A, C, D, and E in the context of urinary tract infections is presented.

## 6. Integrative Strategies in the Prevention and Management of Pediatric Urinary Tract Infections

Emerging evidence underscores the importance of integrating various strategies in the prevention and management of UTI, especially in children [6,67]. Recent research highlights the potential of alternative methods such as probiotics, bladder training, and phytotherapeutic agents, alongside targeted vitamin supplementation, in minimizing UTI and offering complementary support to conventional antibiotic therapies [95,96]. These approaches provide a promising adjunct to address both acute infections and long-term prevention of complications such as renal scarring and recurrence [97]. Dietary factors, e.g., foods rich in fiber and antioxidants, may help maintain a healthy microbiota composition, supporting urinary tract health and enhancing the effects of nutritional interventions, including vitamin supplementation [97]. Probiotics, especially those from the *Lactobacillus* genus, can promote urinary tract health by modulating the microbiota, competitively excluding pathogens, and strengthening the immune response [97]. Both probiotics and, for example, vitamin C may act synergistically by inhibiting pathogen colonization in the urinary tract, making them a promising element of non-antibiotic prevention in children [98,99]. Although using probiotics and vitamins as supportive therapy in pediatric urinary tract infections is promising, potentially improving treatment effectiveness and shortening recovery time, it is still poorly researched and faces significant limitations. The greatest challenge remains the lack of standardization in probiotic preparations and their viability, dosage, and duration of use, which hinders a clear assessment of their clinical efficacy [98,100]. Additionally, the broad age range of children (from infants to adolescents) and thus differences in vitamin dosing also make it difficult to compare study results and draw definitive conclusions [68,100,101,102,103]. Vitamin supplementation in pediatrics is a commonly practiced approach aimed at preventing or treating vitamin deficiencies. In general, its use should be guided by evidence and tailored to the individual needs of each child. A balanced diet remains the best source of vitamins, but supplementation may be necessary in certain situations. Improper use of vitamins, whether in excess or deficiency, can lead to side effects or a lack of therapeutic benefits [104]. The European Food Safety Authority recommends a tolerable upper intake level (UL) for preformed vitamins A, D, and E as well as a population reference intake (PRI) for vitamin C based on age-specific dietary needs to support optimal health and prevent toxicity. Table 1 presents these recommendations categorized by age group. A comprehensive summary of studies evaluating the effectiveness of vitamin supplementation in the treatment of urinary tract infections in children is presented in Table 2 [101,102,103,105].

## 7. Conclusions

The preventive effects of the discussed vitamins manifest in various ways. Vitamin A enhances epithelial repair and integrity, reducing the likelihood of bacterial adhesion and subsequent UTI recurrence. Vitamin C fortifies the immune system through its antioxidant properties, acidifies urine to create a hostile environment for pathogens, and synergistically enhances the effectiveness of antibiotics commonly used to treat urinary tract infections. Vitamin D strengthens innate immunity by promoting the production of antimicrobial peptides such as cathelicidins, which inhibit bacterial colonization, while vitamin E protects against OS, mitigating renal tissue damage and supporting recovery during severe infections. These vitamins not only address nutritional deficiencies but also play pivotal roles in both the prevention and adjunctive management of UTI in pediatric patients.

## Figures and Tables

**Table 1:** Recommended vitamin intake levels and upper limits by age group.

Age Group	Vitamin A UL (µg/Day) [101]	Vitamin C PRI (mg/Day) [105]	Vitamin D UL (µg/Day) [103]	Vitamin E UL (mg/Day) [102]
Infants (4–11 months)	600	20	25	50–60
Children (1–3 years)	800	20	50	100
Children (4–6 years)	1100	30	50	120
Children (7–10 years)	1500	45	50	160
Adolescents (11–14 years)	2000	70	100	220
Adolescents (15–17 years)	2600	100 (boys)/90 (girls)	100	260

Population reference intakes (PRI); tolerable upper intake level (UL).

**Table 2:** Summary of vitamin supplementation studies in the prevention and management of pediatric urinary tract infection.

Age Group	Dosage	Study Group—Age	Study Group	Control Group	Condition	Route	Effectiveness	Ref.
Vitamin A	200,000 IU (single dose)	>12 months	12	12	Recurrent lower urinary tract infections	Oral	Decreased recurrence rates	[34]
	1500 IU/kg/day	1 month to 10 years	15	21	Acute pyelonephritis	Oral	Effective in reducing renal scarring	[45]
	1500 IU/kg/day	2–12 years	36	38	Acute pyelonephritis	Oral	Reduced renal scarring and shorter symptom duration	[35]
	25,000–50,000 IU	1 month to 12 years	25	25	Acute pyelonephritis	Oral	Significantly reduced renal scarring; 20% abnormal DMSA in supplemented group vs. 68% in control group	[43]
Vitamin E	20 IU/kg/day	3 months to 14 years	37	41	Acute pyelonephritis	Oral	No statistically significant reduction in renal scarring but a slight trend toward reduced photopenic areas in girls and children aged 1–3 years	[90]
	20 IU/day	1 month to 10 years	18	21	Acute pyelonephritis	Oral	No worsening of renal lesions; significantly reduced scarring compared to control	[45]
	100 IU	5–12 years	76	76	Acute pyelonephritis	Oral	Reduces acute infection symptoms but does not significantly impact long-term renal scarring	[106]
Vitamin D	1000 IU/day	5–6 years	33	32	Recurrent lower urinary tract infections	Oral	No significant effect in prevention	[74]

Dimercaptosuccinic acid (DMSA).

## Data Availability

Not applicable.

## Abbreviations

The following abbreviations are used in this manuscript:

APN	Acute pyelonephritis
BBD	Bladder bowel dysfunction
CAUTI	Catheter-associated urinary tract infections
DMSA	Dimercaptosuccinic acid
MDA	Malondialdehyde
OS	Oxidative stress
OSI	Oxidative stress index
PRI	Population reference intakes
ROS	Reactive oxygen species
TAS	Total antioxidant status
UL	Tolerable upper intake level
UTI	Urinary tract infections
